# Bacterial Communities Associated with Different *Anthurium andraeanum* L. Plant Tissues

**DOI:** 10.1264/jsme2.ME16099

**Published:** 2016-08-11

**Authors:** Yohanna Sarria-Guzmán, Yosef Chávez-Romero, Selene Gómez-Acata, Joaquín Adolfo Montes-Molina, Eleacin Morales-Salazar, Luc Dendooven, Yendi E. Navarro-Noya

**Affiliations:** 1 CONACYT-Colegio de Postgraduados Campus CampecheChampotón, Campeche, 24450Mexico; 2 ABACUSCinvestav, Mexico City, 07360Mexico; 3 Department of Environmental Engineering, Instituto Tecnológico de CelayaCelaya, Guanajuato, 38010Mexico; 4 Instituto Tecnológico de Tuxtla GutiérrezChiapas, 29050Mexico; 5 CONACYT-Tlaxcala Autonomous UniversityTlaxcala, Tlaxcala, 90000Mexico

**Keywords:** endophytes, endosphere, plant-microbe association, plant microbiome, rhizosphere microbiome

## Abstract

Plant-associated microbes have specific beneficial functions and are considered key drivers for plant health. The bacterial community structure of healthy *Anthurium andraeanum* L. plants was studied by 16S rRNA gene pyrosequencing associated with different plant parts and the rhizosphere. A limited number of bacterial taxa, *i.e.*, *Sinorhizobium*, *Fimbriimonadales*, and *Gammaproteobacteria* HTCC2089 were enriched in the *A. andraeanum* rhizosphere. Endophytes were more diverse in the roots than in the shoots, whereas all shoot endophytes were found in the roots. *Streptomyces*, *Flavobacterium succinicans*, and *Asteroleplasma* were only found in the roots, *Variovorax paradoxus* only in the stem, and *Fimbriimonas* 97%-OTUs only in the spathe, *i.e.*, considered specialists, while *Brevibacillus*, *Lachnospiraceae*, *Pseudomonas*, and *Pseudomonas pseudoalcaligenes* were generalist and colonized all plant parts. The anaerobic diazotrophic bacteria *Lachnospiraceae*, *Clostridium* sp., and *Clostridium bifermentans* colonized the shoot system. Phylotypes belonging to *Pseudomonas* were detected in the rhizosphere and in the substrate (an equiproportional mixture of soil, cow manure, and peat), and dominated the endosphere. *Pseudomonas* included nine 97%-OTUs with different patterns of distribution and phylogenetic affiliations with different species. *P. pseudoalcaligenes* and *P. putida* dominated the shoots, but were also found in the roots and rhizosphere. *P. fluorescens* was present in all plant parts, while *P. resinovorans*, *P. denitrificans*, *P. aeruginosa*, and *P. stutzeri* were only detected in the substrate and rhizosphere. The composition of plant-associated bacterial communities is generally considered to be suitable as an indicator of plant health.

Microorganisms colonize plant surfaces or the ectosphere and live within the plants or endosphere (36). The root-soil interface or rhizosphere is a well-studied part of the ectosphere as is the air-plant interface and phyllosphere (25, 32); however, plant tissues have not been examined in as much detail. Specific biotic and abiotic conditions, and the action of chemical determinants in the plant microenvironments select for different microbial populations (24, 36). They, in turn, have specific functions in the host, such as the suppression of diseases, protection against infections by pathogens, growth stimulation, and promotion of stress resistance, while increasing the mobilization, transport, and uptake of nutrients (1, 9, 22). Therefore, plant-associated microorganisms must be considered as key drivers for plant health and growth.

The rhizosphere favors certain microorganisms from the diverse range of microbes in the surrounding soil (20). Plant-derived exudates and substrates provide the nutrients and the root system a physical niche for rhizospheric microorganisms (14), and the favored microorganism may contribute to plant development. While edaphic soil characteristics are undoubtedly a key determinant of the microbial rhizosphere composition, research has demonstrated that the plant genotype also affects the overall composition of these communities (1, 2, 11).

Although microbes colonizing the plant tissues are generally called endophytes, the endosphere may be divided in the endorhiza (root), anthosphere (flower), spermosphere (seeds), and carposphere (fruit) (38). Bacterial endophytes colonize healthy plant tissue without any disease symptoms. Internalization may result from passive processes, such as through natural openings (stomata and hydathodes), tissue wounds caused by insects and nematodes, root cracks, and germinating radicles, or by active processes such as the production of cell wall degradative enzymes (47). Endophytic bacterial communities are defined by plant characteristics, *e.g.*, species, cultivar, age, health, and developmental stage, and a multitude of abiotic factors, such as soil properties, nutrient status, and climatic conditions (11). Based on the findings of bacterial cultivation studies, which were confirmed by high-throughput sequencing, the majority of the known bacterial endophytic population belongs to *Proteobacteria* (38).

Although the plant microbiota defines microbial diversity, which is important for plant growth, numerous economically important plants, such as crops, ornamental or medicinal plant species, and their relatives have not yet been studied for their associated bacterial communities. Mueller and Sachs (26) suggested that it is possible to transplant “healthy microbiomes” in order to avoid or treat plant diseases in synonymy with what has been found for the gut microbiome. Hence, more basic and practical studies to address the processes leading to community assembly and function in and on healthy plants are needed.

In the present study, we investigated the microbiome of *A. andraeanum* L., an economically important tropical flower (19). Our objectives were i) to describe and compare bacterial communities in the substrate (an equiproportional mixture of soil, cow manure, and peat), rhizosphere, and different parts of *A. andraeanum*, *i.e.*, the roots, stem, leaves, spathe, and spadix, cultivated under controlled conditions, and ii) to identify generalists, *i.e.*, colonizers of all inner tissues of *A. andraeanum*, or specialists, *i.e.*, colonizers of a particular plant organ.

## Materials and Methods

### Plant material

Plantlets of the *A. andraeanum* L. cultivar Sonate were obtained from a commercial farm “Corazón de Meyapac” situated in the municipality of Ocozocoautla de Espinoza, Chiapas, Mexico (16°46′46.90″ N; 93°20′29.84″ W). Plants were grown in 40-L plastic bags containing 15–20 kg substrate, *i.e.*, an equiproportional mixture of soil, cow manure compost, and acidic peat (pH=4.85 and EC [electrolytic conductivity]=120 dS m^−1^) and spaced in rows with 1 m in between and 0.3 m between the bags in the row. Water was applied daily as evapotranspiration was high and the drainage coefficient was 25%. Plants were harvested at developmental stage 6–3 with the spadix ¾ mature (8), placed on ice, and transported to the laboratory.

### DNA extraction and PCR amplification

Endophytic, rhizospheric, and substrate bacterial DNA was isolated from five replicate samples and each sample consisted of three plants. Tissue from the roots, stem, leaves, spathe, and spadix were washed first with tap water and then with sterile distilled water. Tissues were surface disinfected by serially immersing in ethanol 70% (v/v) for 5 min, commercial bleach 2.5% (v/v) for 5 min (roots for 15 min), and rinsed four times with sterile distilled water. The disinfection and elimination of epiphytic bacteria was confirmed with the absence of amplification of the 16S rRNA and 18S rRNA genes using water for the final wash as a template. Liquid nitrogen was applied to 2 g of plant tissue and ground with a sterile mortar. The ground tissues were added to 15-mL propylene tubes and 2 mL 10 mM Tris-HCl (pH 8) plus 160 μL 10 mg mL^−1^ lysozyme. The mixture was kept at 37°C for 1 h. The cells were lysed with 2 mL sodium dodecyl sulfate (SDS) 10% (w/v). Sterile glass beads were added and mixed on a vortex for 10 min. Proteins were eliminated and DNA precipitated as described by Navarro Noya *et al.* (27). Substrate (1.5 g) and rhizosphere DNA (1.5 g soil firmly adhered to the roots) was extracted with three different methods. A sub-sample of 0.5 g of the substrate and rhizosphere was used for each extraction technique and pooled as described previously (31). The first technique consisted of the enzymatic digestion of the bacterial cell walls, the second with chemical (with sodium dodecyl sulphate) and physical rupture of the cells, and the third with chemical (with Triton X-100) and physical rupture of the cells (31).

The primers 8-F and 556-R were used to amplify the variable regions V1–V3 of the 16S rRNA bacterial genes. The PCR mixture and thermal cycling were performed as described by Navarro-Noya *et al.* (27). All samples were amplified in triplicate, pooled in equal concentrations, and purified using the DNA clean and concentrator purification kit as recommended by the manufacturer (Zymo Research, Irvine, CA, USA). Quantification of the PCR products was done using the PicoGreen dsDNA assay (Invitrogen, Carlsbad, USA) and a NanoDrop 3300 fluorospectrometer (Thermo Fisher Scientific, Waltham, MA, USA). Amplicon libraries were sequenced with a Roche GS-FLX Titanium 454 pyrosequencer (Roche, Mannheim, Germany) by Macrogen DNA Sequencing Service (Seoul, Korea).

### Bioinformatics and statistical analysis

Sequences were processed through the QIIME pyrosequencing pipeline (http://www.qiime.org/) (3). Sequences of low quality were removed and noise from the sequences was eliminated with Denoiser (33). The screened sequences were used to determine operational taxonomic units (OTUs) with open-reference clustering using Uclust at a similarity threshold of 97% (97%-OTUs) (10). Taxonomic assignations were performed with the RDP classifier 2.2 at an 80% confidence threshold (48) and based on the Greengenes reference database (version 1210) with one representative sequence of each 97%-OTU. Sequences belonging to 16S rRNA of the chloroplast from the plant were eliminated. Diversity and species richness estimators were calculated within QIIME (3). Significant differences in alpha-diversity parameters and the abundance of the bacterial groups were calculated with the general linear model procedure (GLM, 39).

The representative 97%-OTUs sequences were aligned with the Greengenes core-set-aligned available at http://greengenes.lbl.gov/ at a minimum sequence identity of 75% using PyNAST (4). A maximum-likelihood phylogenetic tree was constructed with the aligned sequences using FastTree 2.1.3 (29). A UniFrac distance matrix was generated using phylogenetic information and occurrence data to compare bacterial communities associated with the inner *A. andraeanum* tissues (endosphere), rhizosphere, and found in the substrate. A multivariate analysis, *i.e.*, principal coordinate analysis (PCoA), was applied using the UniFrac distance matrix to examine and visualize dissimilarities in bacterial communities in the endosphere, rhizosphere, and substrate of *A. andraeanum*. A permutational multivariate analysis of variance (perMANOVA) was performed using UniFrac pairwise distances to test significant differences between bacterial communities (*n* = 999). Significant differences in the abundance of taxonomic groups as a result of the different treatments were calculated using an analysis of variance (ANOVA) based on the minimum significant difference using the general linear model procedure (GLM, 39). Sequences belonging to the five replicates were combined to schematize bacterial distributions at the family and 97%-OTU level in the inner tissues of the *A. andraeanum*, rhizosphere and substrate.

### Data accessibility

The 35 pyrosequencing-derived 16S rRNA gene sequence datasets were submitted to the NCBI Sequence Read Archive (SRA) under the BioProject accession number PRJNA315163.

## Results

### Bacterial diversity and composition of microenvironments of *A. andraeanum*

**Fig. 1:**
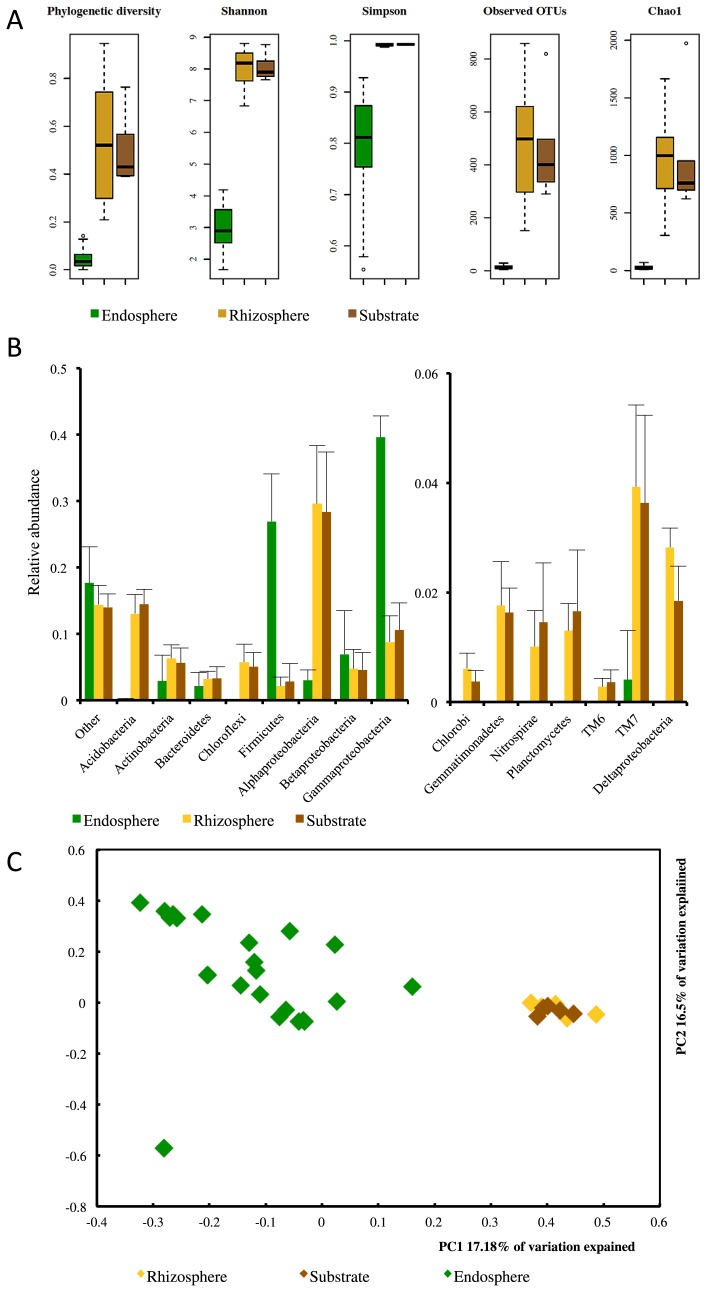
Bacterial communities associated with the rhizosphere and endosphere of *Anthurium andraeanum* L. plants and the substrate, *i.e.*, a mixture of soil, compost, and acidic peat. Boxplots of bacterial diversity (Phylogenetic diversity, Shannon and Simpson indices) and species richness (observed 97%-OTUs and Chao1 richness estimator) (A), relative abundance of bacterial phyla and four classes of *Proteobacteria* (bars indicate standard deviation *n* = 5) (B), and principal coordinate analyses of the UniFrac distances of bacterial communities (C).

A total of 13,160 denoised and high quality sequences were included in the bacterial communities analysis and 2,517 OTUs at a 97% similarity threshold (97%-OTUs) were detected (Table S1). Species richness and diversity were higher in the rhizosphere and substrate than in the plant (Fig. 1A). Diversity and richness were not significantly different between the substrate and rhizosphere, but varied more in the latter than in the former (Table 1).

Nine different phyla were detected in the endosphere and 26 in the substrate and rhizosphere (Fig. 1B). *Proteobacteria* (39.8%, mainly *Gammaproteobacteria*), *Firmicutes* (26.9%), and *Actinobacteria* (2.9%) dominated in the endosphere. *Proteobacteria* (25.3%, mainly *Alphaproteobacteria*), *Acidobacteria* (11.8%), and *Chloroflexi* (6.4%) dominated in the rhizosphere, while *Proteobacteria* (24.5%, mainly *Alphaproteobacteria*), *Acidobacteria* (14.4%), and *Actinobacteria* (5.2%) dominated in the substrate.

The PCoA of UniFrac distances showed that the bacterial community inside the plants was different from those in the rhizosphere and substrate (perMANOVA 999, pseudo-*F*: 3.4374, *P* value: 0.001) (Fig. 1C). Although the relative abundances of *Sinorhizobium*, *Fimbriimonadales*, and *Gammaproteobacteria* HTCC2089 were significantly higher in the rhizosphere than in the substrate, the bacterial communities of the rhizosphere and substrate were not significantly different (perMANOVA 999, pseudo-*F*: 0.4725, *P* value: 1.00) (Table 2).

Phylotypes of *Rhizobium* were found in the substrate and plant, but not in the rhizosphere. Phylotypes belonging to *Alkalibacterium*, *Xanthomonas*, and *Asteroleplasma* were only found in the endosphere (Table S2). *Flavisolibacter*, *Caldilinea*, *Haloplasmataceae*, and *Thermobacillus* were unique to the rhizosphere, while *Candidatus Solibacter*, *Ornithinibacillus*, *Ureibacillus*, and *Curvibacter* were only found in the substrate. *Microbacterium*, *Streptomyces*, *Flavobacterium*, *Sinorhizobium*, *Bradyrhizobium*, *Azoarcus*, *Halomonas*, and *Pseudomonas* were found in the substrate, rhizosphere, and plants.

### Bacterial diversity of the endophytic community in tissues of *A. andraeanum*

**Fig. 2:**
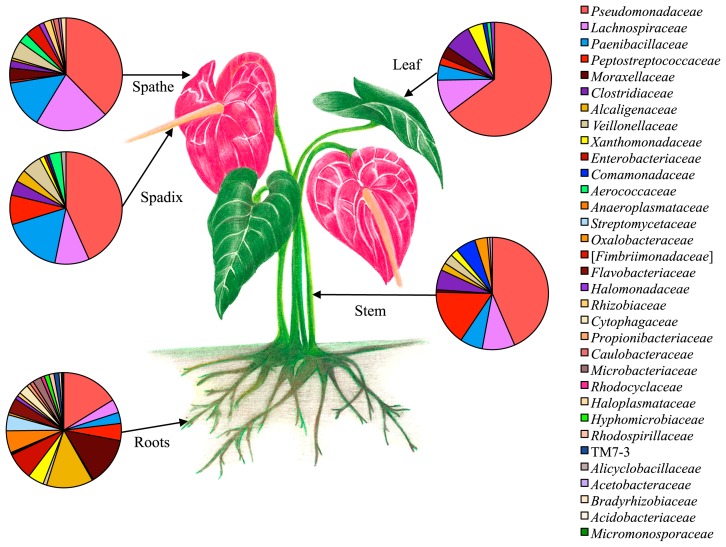
Relative abundance of bacterial endophytic families associated with roots, stem, leaves, spathe, and spadix of healthy *Anthurium andraeanum* L. plants.

Thirty-three bacterial families were found in the stem, leaves, spadix, spathe, and roots of *A. andraeanum* (Fig. 2, Table S3). Family richness was in the order of roots (29 families) > spathe (16) > stem (13) > spadix (12) > leaves (10), with *Pseudomonadaceae* being the most abundant inside the plant.

**Fig. 3:**
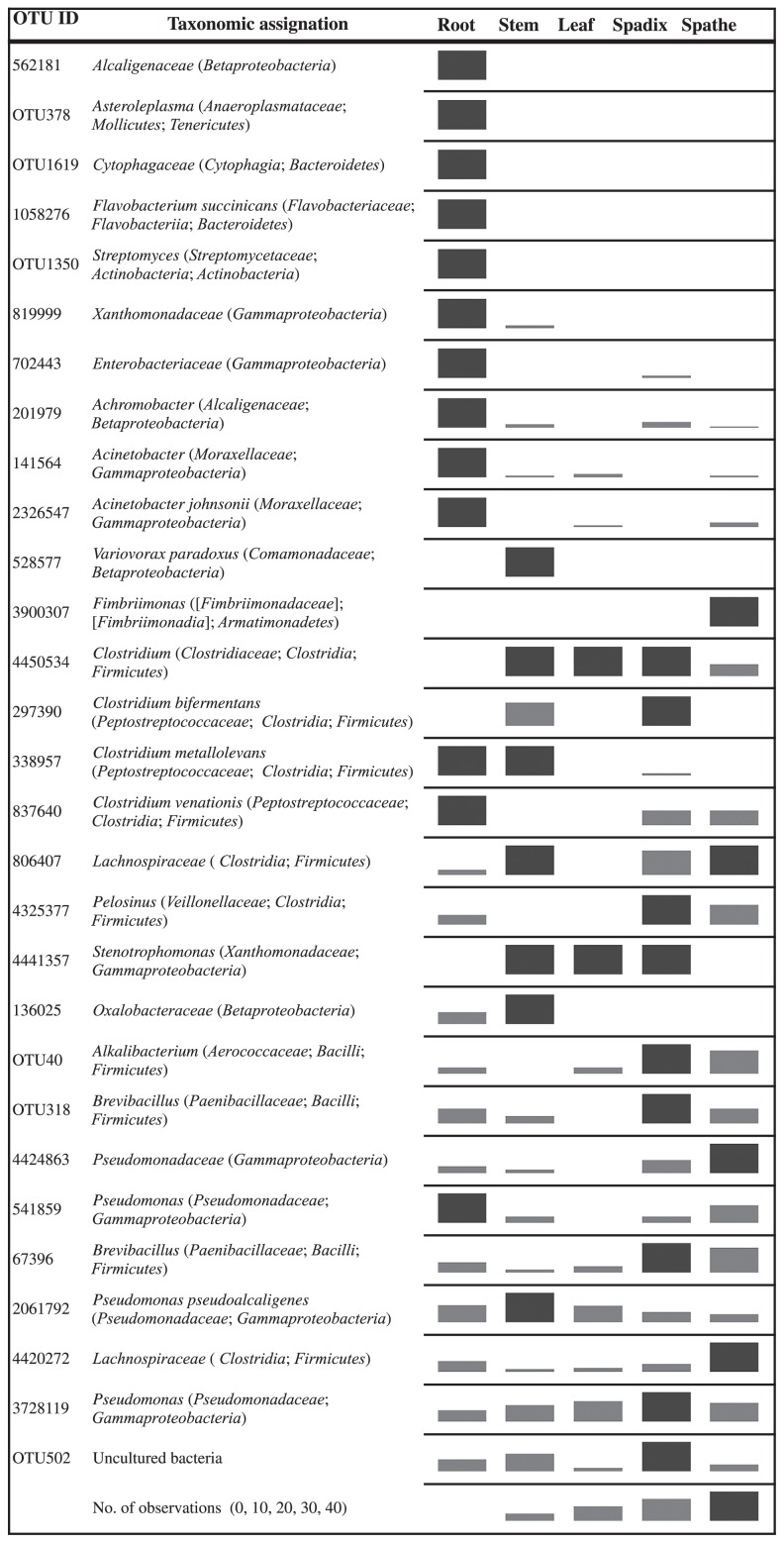
Frequency of bacterial endophytic operational taxonomic units at 97% similarity (97%-OTU) associated with roots, stem, leaves, spathe, and spadix of healthy *Anthurium andraeanum* L. plants.

Fig. 3 illustrates the distribution of the most abundant endophytic 97%-OTUs in *A. andraeanum*. The 97%-OTUs found in all plant parts belonged to *Brevibacillus*, *Lachnospiraceae*, *Pseudomonas*, and *P. pseudoalcaligenes*. Most of the 97%-OTUs detected in the roots were found in the above ground parts of the plants, except for OTU_4450534 (*Clostridium* and *Clostridiaceae*), OTU_297390 (*C. bifermentans* and *Pestostreptococaceae*), OTU_528577 (*Variovorax paradoxus*) and OTU_4441357 (*Stenotrophomonas*). OTU_1350 (*Streptomyces*), OTU_1619 (*Cytophagaceae*), OTU_1058276 (*Flavobacterium succinicans*), and OTU_378 (*Asteroleplasma*) were only found in the roots, OTU_528577 (*V. paradoxus*) only in the stem, and OTU_3900307 (*Fimbriimonas*) only in the spathe. Table S4 exhibits the relative abundance of all the genera detected in the inner tissues and rhizosphere of *A. andraeanum*.

### Distribution of *Pseudomonadaceae*

**Fig. 4:**
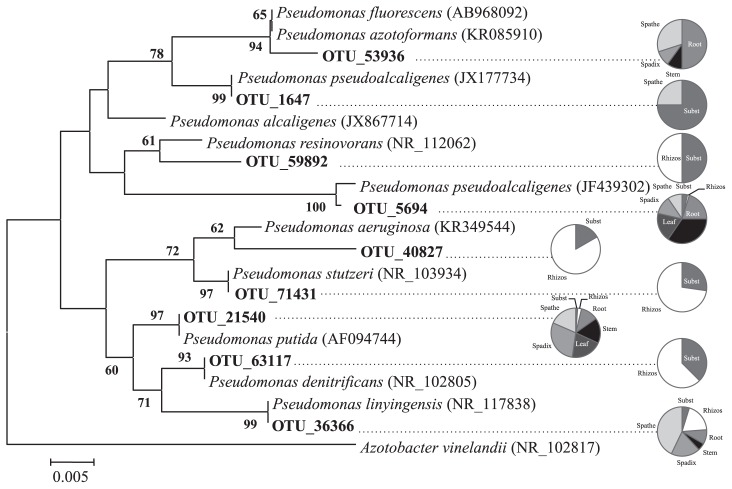
Neighbor joining phylogenetic tree based on 16S rRNA partial gene sequences showing relationships between operational taxonomic units at 97% cut–off (97%-OTU) and sequences of databases of *Pseudomonas* spp. Numbers in parenthesis are the GenBank accession numbers. The scale bar indicates the nucleotide substitutions in each site. *Azotobacter vinelandii*^T^ (NR102817) served as an outgroup. Pie charts indicate the proportion of observations of 97%-OTU in the different tissues of healthy *Anthurium andraeanum* L. plants, rhizosphere, and the substrate, *i.e.*, a mixture of soil, compost, and acidic peat. Subst = substrate; Rhizos = rhizosphere.

*Pseudomonadaceae* was the most abundant endophytic bacterial family, with nine 97%-OTUs. They were affiliated with *P. fluorescens*, *P. pseudoalcaligenes*, *P. resinovorans*, *P. putida*, *P. linyngensis*, *P. denitrificans*, *P. aeruginosa*, and *P. stutzeri* (Fig. 4). *P. resinovorans*, *P. aeruginosa*, *P. denitrificans*, and *P. stutzeri* were only found in the substrate and rhizosphere, while *P. fluorescens* was only found in the endosphere. The most abundant 97%-OTUs inside the plant, *i.e.*, *P. pseudoalcaligenes* and *P. putida*, were also found in the rhizosphere and substrate.

## Discussion

Plants are in constant contact with microorganisms, some of which are able to colonize the plant and survive (32). A growing body of research shows that plant-associated microbes contribute to the health of the host and increase its resistance to biotic and abiotic stresses (2). A recently proposed technique, *i.e.*, host-mediated microbiome selection, has suggested the selection of artificially microbiomes to improve plant fitness (26). However, this new technology requires a better understanding of plant physiology and the microbial community associated with healthy plants. In the present study, we describe the rhizospheric and endophytic communities of healthy *A. andraeanum* plants and examined the patterns of distribution, whether specialist or generalist, of endophytic bacteria within the plant.

The endosphere is a special habitat and endophytes must interact successfully with the plant to survive (1); thus, bacterial diversity was lower in the plant than in the substrate or rhizosphere (Fig. 1A). Additionally, endophytes reduce the entrance of other microorganisms, including pathogens, through the induction of plant defense mechanisms, *i.e.*, the induced systemic resistance (ISR) (17).

Rhizosphere-associated microbes originate from the surrounding substrate and are often directly or indirectly involved in plant growth promotion (20). The two main factors shaping root-associated bacterial communities are edaphic and plant characteristics (2). A limited number of bacterial taxa were enriched in the rhizosphere of *A. andraeanum*, *i.e.*, *Sinorhizobium*, *Fimbriimonadales*, and the high-throughput culture collection (HTCC) clade 2089 of *Gammaproteobacteria* (Table 2). *Sinorhizobium* is best known for its capacity to fix atmospheric N_2_ in a symbiotic relationship with leguminous plants, but it also increases the shoot and root biomass (12). Members of *Gammaproteobacteria* HTCC2089 are marine oligotrophic bacteria (6) and are associated with corals and sponges (7). Although it is difficult to speculate why they were more abundant in the rhizosphere than in the substrate, nutrient uptake by the plant may create oligotrophic microhabitats favoring strains of HTCC2089.

Root endophytes were more diverse than those in the above ground parts of *A. andraeanum* and nearly all root endophytes were also found in the shoots (Fig. 3). Entry through root cracks is the main portal for bacterial colonization (38). Bacterial taxa exhibited different patterns of distribution inside the different organs. *Brevibacillus* was found in all plant parts. Several studies identified *Brevibacillus* as an endophyte of alfalfa (*Medicago sativa* L.), banana (*Musa* sp. L.), and saffron (*Crocus sativus* L.) (41, 43, 46). Phylotypes belonging to this genus have the capacity to promote plant growth through the production of siderophores and indole-3 acetic acid and fix nitrogen (13, 28). They exhibit ACC (1-Aminocyclopropane-1-Carboxylate) deaminase activity and contribute to the arbuscular mycorrhizae association (28, 35).

*Streptomyces* 97%-OTUs were only detected in the roots of *A. andraeanum*. *Streptomyces* spp. contain well-known endophyte strains and have often been isolated from different crops and medicinal or woody plants (18, 40). Their ecological niche as endophytes is mostly as biocontrol agents that produce antimicrobial substances to inhibit other microorganisms (15). They have biotechnological potential as an important source of bioactive secondary metabolites, such as antimicrobials (44). *Asteroleplasma* (*Anaeroplasmataceae*, Anaeroplasmatales, Mollicutes), a strict anaerobic anaeroplasma, was also found inside the roots. They are assumed to have originated from the substrate that contained 33% composted cow manure because anaeroplasmas have been found in ovine and bovine rumens (49). Mollicutes have been found in the early stages of composting and vermicomposting of cow manure (34).

Anaerobic bacteria, such as *Lachnospiraceae*, *Clostridium* (*Clostridiaceae*), and *Clostridium bifermentans* (*Pestostreptococaceae*) were found to colonize all shoots parts, *C. metallolevans* (*Pestostreptococaceae*) and *C. venationis* (*Pestostreptococaceae*) were detected in the roots and spathe/spadix, and OTU4420272 *Lachnospiraceae* was found in all tissues (Fig. 3). Nitrogen-fixing strains of these taxa have been isolated and/or detected in several species of *Gramineae*, *Leguminosae*, *Polygonaceae*, *Rhizophoraceae*, *Solanaceae*, and *Verbenaceae* (23, 24, 37). This study confirms the wide distribution of endophytic *Clostridia*. *Clostridium* are spore forming and one of the major cellulolytic/ pectinolytic and fermentative bacterial groups (5, 45). Additionally, Minamisawa *et al.* (23) suggested that plant-dwelling clostridia proliferate in anoxic microzones as a result of bacterial activity or plant respiration, while they survive as spores when O_2_ concentrations are higher.

Nine 97%-OTUs of *Pseudomonas* were found in the three microenvironments studied, *i.e.*, the endosphere, rhizosphere, and substrate and dominated inside the tissues of all the organs. *Pseudomonas*, together with *Bacillus*, are considered a model for the study of plant-microbe interactions (30). The importance of *Pseudomonas* for plants has been corroborated in different studies, *i.e.*, culture-dependent, molecular fingerprint methods, and metagenomics (22). The phylogenies of the nine 97%-OTUs belonging to *Pseudomonas* were analyzed and different distribution patterns emerged (Fig. 4). The 97%-OTU 5694 affiliated with *P. pseudoalcaligenes* and 97%-OTU 21540 with *P. putida* were dominant in the above ground parts of *A. andraeanum*, but were also found in the roots and rhizosphere (Fig. 4). The 97%-OTUs 59892, 63117, 40827, and 71431 were phylogenetically related to *P. resinovorans*, *P. denitrificans*, *P. aeruginosa*, and *P. stutzeri*, respectively, and were only retrieved from the substrate and rhizosphere. The 97%-OTUs 53936 affiliated with *P. fluorescens* were found throughout the plant, while two species dominated in the shoot tissues, *i.e.*, *P. pseudoalcaligenes* and *P. putida*. The plant-microbe interactions with *Pseudomonas* (*sensu stricto*) are complex because strains of this genus have versatile metabolic capacities and occupy different niches. On one hand, some strains produce plant growth promoters and suppress a wide range of phytopathogens through the production of antimicrobials or secretion of signaling molecules, such as lipoproteins, phenazime-1-carboxylic acid, and 2,4-DAPG (20, 30, 50). On the other hand, several strains of *P. syringae* are well-known phytopathogens or pathovars that cause important economic losses. *Pseudomonas* also contains soil- or rhizosphere-borne species associated with human diseases, and many other opportunistic pathogen species (21). No *Pseudomonas* species with a history of phytopathogenicity were detected in *A. andraeanum*. The different patterns of distribution of the *Pseudomonas* 97%-OTUs related may be explained by 1) the highly competitive niches generated by the nutrient-enriched environments in the rhizosphere and plant tissues, or 2) the differential genetic homogeneity and heterogeneity within *Pseudomonas* species. For example, comparisons of >1,000 *Pseudomonas* genomes based on pangenome and core genome analyses revealed that *P. aeruginosa* genomes were a homogenous cluster distinct from other *Pseudomonas* species, while the genomes of *P. fluorescens* were highly diverse and heterogenic (16, 42).

## Conclusion

Different parts of *A. andraeanum* had different endophytic communities, with the largest diversity being detected in the roots. The main entry of bacterial endophytes appears to be the roots because most endophytes found in the above ground parts were found also in the roots. Several *Pseudomonas* spp. were identified and showed different patterns of distribution inside *A. andraeanum*, in the rhizosphere and in the substrate. We suggest that bacteria found in healthy plants are suitable as indicators of plant health.

## Supplementary Information



## Figures and Tables

**Table 1:** Comparison of bacterial richness and diversity of communities in the substrate, rhizosphere, and endosphere of *Anthurium* and inside tissues

Comparison roots *vs* stem *vs* leaves *vs* spathe *vs* spadix			

Alpha-diversity parameter^a^	MSD^b^	*F* value	*P* value
Phylogenetic diversity	0.06	2.39	0.0913
Simpson	0.15	5.12	0.0052
Shannon	0.88	8.41	0.0009
Observed OTU	8.75	7.26	0.0009
Chao1	29.05	3.23	0.0339

Comparison endosphere *vs* rhizosphere *vs* compost			

Phylogenetic diversity	0.16	51.87	<0.0001
Simpson	0.12	17.43	<0.0001
Shannon	0.90	200.30	<0.0001
Observed OTU	163.66	50.69	<0.0001
Chao1	354.75	45.93	<0.0001

a Simpson: Simpson diversity index, Shannon: Shannon-Weaver diversity index, OTU: Operational taxonomic units defined at 97% similarity with UCLUST, Chao1: bias corrected Chao1.

b MSD: Minimum significant difference.

**Table 2:** Bacterial taxa significantly enriched in the rhizosphere of *Anthurium* (*P*<0.05)

Bacterial taxa	Mean (%)		MSD^a^	*F* value	*P* value

	Rhizosphere	Substrate			
*Fimbriimonadales* (*Fimbriimonadia*; *Armatimonadetes*)	0.189	0	0.0017	6.60	0.0326
*Sinorhizobium* (*Rhizobiaceae*; *Rhizobiales*; *Alphaproteobacteria*)	0.431	0.017	0.0021	21.18	0.0017
HTCC2089 (HTCC2188; *Gammaproteobacteria*)	0.352	0.099	0.0021	7.41	0.0262

a MSD: Minimum significant difference at the *P*<0.05 level
