# Supplementary material for: Bacterial Communities Associated with Different Anthurium andraeanum L. Plant Tissues
Source: Microbes Environ. 2016 Aug 11;31(3):321–8. doi: 10.1264/jsme2.ME16099 (PMC5017810; doi:10.1264/jsme2.ME16099)
Supplement: Supplementary file 1 [file 31_321_s1.pdf]

Table S1 Summary of samples and pyrosequencing data analysis.

Sample	Barcode sequence	Number of high quality reads[a]	Number of reads after denoising	Number of reads after filter[b]
Substrate	TGCTCGCTAC	1207	1086	1080
	ACGTGCAGCG	579	463	463
	ACTCACAGAG	1170	1053	1033
	AGACTCAGCG	2189	1602	1502
	AGAGAGTGTG	776	641	541
Rhizosphere	AGCTATCGCG	1640	1558	1467
	AGTCTGACTG	1342	1168	1160
	AGTGAGCTCG	384	354	244
	ATAGCTCTCG	679	624	504
	ATCACGTGCG	2263	2215	2196
Roots	CTATAGCGTA	3690	3321	112
	TATGCTAGTA	2702	2162	88
	TGAGTCAGTA	477	334	102
	ACAGTCGTGC	3166	2533	84
	ACTCGCGCAC	3330	3164	107
Stem	CCTACGCTCTA	5232	4552	172
	TCACGCGAGA	4747	4272	166
	TGTAGTGTGA	2157	1726	157
	ACATGACGAC	2170	1953	106
	AGAGCGTCAC	3031	2122	131
Leaves	TACGTCATCA	2327	1862	111
	TCACGCGAGA	2746	2581	101
	TGTAGTGTGA	2725	2453	122
	ACATGACGAC	3503	2802	103
	AGAGCGTCAC	2836	1985	130
Spathe	TAGTCGCATA	3180	2544	128
	TCGCTGCGTA	964	916	105
	TGTCGTCGCA	1515	1318	98
	ACGTCTCATC	3643	3279	115
	AGTAGTGATC	2256	1805	132
Spadix	TATATATACA	5029	4526	109
	TCTGACGTCA	4498	3149	122
	ACACATACGC	2770	2216	120
	ACTCATCTAC	5097	4842	99
	AGTGACACAC	1321	1149	150
Total count		87341	74100	13160

[a] Reads at high quality i.e. >200 bp in length, > 25 phred quality threshold and containing any unresolved nucleotides. [b] Datasets without sequences belonged to 16S rRNA of the chloroplast from the plant

Table S2 Relative abundance of bacterial genus found in the substrate, and associated with the rhizosphere and endosphere of healthy *Anthurium andraeanum* L. plants.

Taxon	Relative abundance (%)		
	Substrate	Rhizosphere	Endosphere
Firmicutes; Bacilli; Lactobacillales; Aerococcaceae; *Alkalibacterium*	0	0	1.156
Firmicutes; Clostridia; Clostridiales; Clostridiaceae; *Alkaliphilus*	0	0	0.420
Firmicutes; Clostridia; Clostridiales; Veillonellaceae; *Pelosinus*	0	0	2.313
Firmicutes; Bacilli; Haloplasmatales; Haloplasmataceae	0	0	1.197
Proteobacteria; Gammaproteobacteria; Xanthomonadales; Xanthomonadaceae; *Xanthomonas*	0	0	0.420
Tenericutes; Mollicutes; Anaeroplasmatales; Anaeroplasmataceae; *Asteroleplasma*	0	0	1.892
Actinobacteria; Actinobacteria; Actinomycetales; Propionibacteriaceae; *Propionibacterium*	0	0.031	0.525
Firmicutes; Clostridia; Clostridiales; Peptostreptococcaceae; *Clostridium*	0	0.015	3.259
Proteobacteria; Betaproteobacteria; Burkholderiales; Alcaligenaceae; *Achromobacter*	0	0.031	3.785
Proteobacteria; Gammaproteobacteria; Xanthomonadales; Xanthomonadaceae; *Stenotrophomonas*	0	0.031	0.630
Actinobacteria; Actinobacteria; Actinomycetales; Cellulomonadaceae; *Demequina*	0	0.031	0
Actinobacteria; Actinobacteria; Actinomycetales; Nocardiopsaceae; *Thermobifida*	0	0.047	0
Bacteroidetes; [Saprospirae]; [Saprospirales]; Chitinophagaceae; *Flavisolibacter*	0	0.031	0
Chloroflexi; Anaerolineae; Caldilineales; Caldilineaceae; *Caldilinea*	0	0.047	0
Firmicutes; Bacilli; Bacillales; Paenibacillaceae; *Thermobacillus*	0	0.063	0
Firmicutes; Bacilli; Bacillales; Thermoactinomycetaceae; *Laceyella*	0	0.031	0
Firmicutes; Clostridia; Clostridiales; Clostridiaceae; *Oxobacter*	0	0.079	0
GN02; GKS2-174	0	0.031	0
Planctomycetes; Planctomycetia; Planctomycetales; Planctomycetaceae; *Planctomyces*	0	0.127	0
Proteobacteria; Alphaproteobacteria; Caulobacterales; Caulobacteraceae; *Asticcacaulis*	0	0.047	0
Proteobacteria; Alphaproteobacteria; Rhizobiales; Methylocystaceae; *Pleomorphomonas*	0	0.047	0
Proteobacteria; Alphaproteobacteria; Sphingomonadales; Sphingomonadaceae; *Novosphingobium*	0	0.079	0
Proteobacteria; Deltaproteobacteria; Bdellovibrionales; Bdellovibrionaceae; *Bdellovibrio*	0	0.031	0
Proteobacteria; Deltaproteobacteria; Myxococcales; Nannocystaceae; *Plesiocystis*	0	0.111	0
Verrucomicrobia; [Spartobacteria]; [Chthoniobacterales]; [Chthoniobacteraceae]; DA101	0	0.063	0
WS3; PRR-12; Sediment-1; CV106;	0	0.031	0
Proteobacteria; Alphaproteobacteria; Rhizobiales; Rhizobiaceae; *Rhizobium*	0.016	0	0.315
Acidobacteria; Solibacteres; Solibacterales; Solibacteraceae; Candidatus *Solibacter*	0.033	0	0
Armatimonadetes; 0319-6E2	0.033	0	0
Bacteroidetes; Sphingobacteriia; Sphingobacteriales; Sphingobacteriaceae; *Solitalea*	0.050	0	0
BRC1; PRR-11	0.033	0	0
Firmicutes; Bacilli; Bacillales; Bacillaceae; *Ornithinibacillus*	0.033	0	0
Firmicutes; Bacilli; Bacillales; Planococcaceae; *Ureibacillus*	0.084	0	0
Firmicutes; Bacilli; Bacillales; Thermoactinomycetaceae; *Thermoactinomyces*	0.033	0	0
GOUTA4	0.067	0	0
OD1	0.033	0	0
OP11; WCHB1-64	0.033	0	0
Planctomycetes; Planctomycetia; Pirellulales; Pirellulaceae; A17	0.033	0	0
Proteobacteria; Alphaproteobacteria; Sphingomonadales; Erythrobacteraceae; *Altererythrobacter*	0.033	0	0
Proteobacteria; Betaproteobacteria; Burkholderiales; Comamonadaceae; *Curvibacter*	0.067	0	0
Proteobacteria; Gammaproteobacteria; [Marinicellales]; [Marinicellaceae]	0.084	0	0
Verrucomicrobia; [Spartobacteria]; [Chthoniobacterales]; [Chthoniobacteraceae]	0.033	0	0
Actinobacteria; Actinobacteria; Actinomycetales; Microbacteriaceae; *Microbacterium*	0.067	0.063	0.736
Actinobacteria; Actinobacteria; Actinomycetales; Streptomycetaceae; *Streptomyces*	0.050	0.222	1.366
Armatimonadetes; [Fimbriimonadia]; [Fimbriimonadales]; [Fimbriimonadaceae]; *Fimbriimonas*	0.777	0.413	0.630
Bacteroidetes; Flavobacteriia; Flavobacteriales; Flavobacteriaceae; *Flavobacterium*	0.473	0.524	1.261
Firmicutes; Bacilli; Bacillales; Alicyclobacillaceae; *Alicyclobacillus*	0.050	0.015	0.210
Firmicutes; Bacilli; Bacillales; Paenibacillaceae; *Brevibacillus*	0.067	0.095	6.203
Firmicutes; Bacilli; Bacillales; Paenibacillaceae; *Paenibacillus*	0.067	0.031	0.630
Firmicutes; Clostridia; Clostridiales; Clostridiaceae; *Clostridium*	0.033	0.015	1.997
Firmicutes; Clostridia; Clostridiales; Peptostreptococcaceae; [*Clostridium*]	0.033	0.095	2.103
Proteobacteria; Alphaproteobacteria; Rhizobiales; Bradyrhizobiaceae; *Bradyrhizobium*	0.355	0.142	0.210
Proteobacteria; Alphaproteobacteria; Rhizobiales; Hyphomicrobiaceae; *Devosia*	0.811	1.191	0.315
Proteobacteria; Alphaproteobacteria; Rhizobiales; Rhizobiaceae; *Sinorhizobium*	0.016	0.524	0.315
Proteobacteria; Betaproteobacteria; Burkholderiales; Comamonadaceae; *Acidovorax*	0.016	0.031	0.210
Proteobacteria; Betaproteobacteria; Burkholderiales; Comamonadaceae; *Ramlibacter*	0.067	0.047	0.105
Proteobacteria; Betaproteobacteria; Burkholderiales; Comamonadaceae; *Variovorax*	0.084	0.047	0.841
Proteobacteria; Betaproteobacteria; Rhodocyclales; Rhodocyclaceae; *Azoarcus*	0.084	0.031	0.105
Proteobacteria; Gammaproteobacteria; Oceanospirillales; Halomonadaceae; *Halomonas*	0.388	0.413	0.630
Proteobacteria; Gammaproteobacteria; Pseudomonadales; Moraxellaceae; *Acinetobacter*	0.338	0.063	5.047
Proteobacteria; Gammaproteobacteria; Pseudomonadales; Pseudomonadaceae; *Pseudomonas*	0.490	0.571	27.339
Actinobacteria; Actinobacteria; Actinomycetales; Microbacteriaceae; *Agromyces*	0.169	0.063	0
Actinobacteria; Actinobacteria; Actinomycetales; Microbacteriaceae; *Cryocola*	0.067	0.015	0
Actinobacteria; Actinobacteria; Actinomycetales; Mycobacteriaceae; *Mycobacterium*	0.371	0.301	0
Actinobacteria; Actinobacteria; Actinomycetales; Nocardioidaceae; *Nocardioides*	0.236	0.158	0
Actinobacteria; Actinobacteria; Actinomycetales; Promicromonosporaceae; *Xylanimicrobium*	0.016	0.015	0

Table S2 (continued)

Taxon	Substrate	Rhizosphere	Endosphere
Actinobacteria; Actinobacteria; Actinomycetales; Pseudonocardiaceae; *Saccharomonospora*	0.016	0.031	0
Actinobacteria; Actinobacteria; Actinomycetales; Williamsiaceae; *Williamsia*	0.033	0.031	0
Actinobacteria; Thermoleophilia; Solirubrobacterales; Conexibacteraceae; *Conexibacter*	0.135	0.063	0
Bacteroidetes; [Saprospirae]; [Saprospirales]; Chitinophagaceae; *Flavihumibacter*	0.033	0.031	0
Bacteroidetes; Cytophagia; Cytophagales; Cytophagaceae; *Adhaeribacter*	0.050	0.047	0
Firmicutes; Bacilli; Bacillales; Bacillaceae; *Bacillus*	1.690	1.064	0
Firmicutes; Bacilli; Bacillales; Planococcaceae; *Lysinibacillus*	0.033	0.047	0
Firmicutes; Bacilli; Bacillales; Planococcaceae; *Solibacillus*	0.118	0.158	0
Firmicutes; Bacilli; Bacillales; Thermoactinomycetaceae; *Planifilum*	0.439	0.222	0
Nitrospirae; Nitrospira; Nitrospirales; Nitrospiraceae; *Nitrospira*	1.183	0.841	0
Proteobacteria; Alphaproteobacteria; Caulobacterales; Caulobacteraceae; *Phenylobacterium*	0.304	0.428	0
Proteobacteria; Alphaproteobacteria; Rhizobiales; Bradyrhizobiaceae; *Balneimonas*	0.067	0.142	0
Proteobacteria; Alphaproteobacteria; Rhizobiales; Hyphomicrobiaceae; *Hyphomicrobium*	0.422	0.683	0
Proteobacteria; Alphaproteobacteria; Rhizobiales; Hyphomicrobiaceae; *Pedomicrobium*	0.405	0.333	0
Proteobacteria; Alphaproteobacteria; Rhizobiales; Hyphomicrobiaceae; *Rhodoplanes*	6.729	6.973	0
Proteobacteria; Alphaproteobacteria; Rhizobiales; Methylobacteriaceae; *Methylobacterium*	0.185	0.158	0
Proteobacteria; Alphaproteobacteria; Rhizobiales; Phyllobacteriaceae; *Mesorhizobium*	0.490	0.317	0
Proteobacteria; Alphaproteobacteria; Rhizobiales; Rhizobiaceae; *Agrobacterium*	0.050	0.095	0
Proteobacteria; Alphaproteobacteria; Rhizobiales; Rhodobiaceae; *Afifella*	0.067	0.031	0
Proteobacteria; Alphaproteobacteria; Rhizobiales; Xanthobacteraceae; *Labrys*	0.033	0.015	0
Proteobacteria; Alphaproteobacteria; Rhodobacterales; Rhodobacteraceae; *Amaricoccus*	0.050	0.095	0
Proteobacteria; Alphaproteobacteria; Sphingomonadales; Sphingomonadaceae; *Sphingobium*	0.101	0.158	0
Proteobacteria; Alphaproteobacteria; Sphingomonadales; Sphingomonadaceae; *Sphingopyxis*	0.033	0.047	0
Proteobacteria; Betaproteobacteria; Burkholderiales; Burkholderiaceae; *Burkholderia*	0.050	0.063	0
Proteobacteria; Betaproteobacteria; Burkholderiales; Comamonadaceae; *Methylibium*	0.050	0.047	0
Proteobacteria; Betaproteobacteria; Burkholderiales; Comamonadaceae; *Rubrivivax*	0.422	0.365	0
Proteobacteria; Betaproteobacteria; Burkholderiales; Oxalobacteraceae; *Cupriavidus*	0.118	0.047	0
Proteobacteria; Betaproteobacteria; Burkholderiales; Oxalobacteraceae; *Ralstonia*	0.033	0.079	0
Proteobacteria; Gammaproteobacteria; Legionellales; Coxiellaceae; *Aquicella*	0.185	0.238	0
Proteobacteria; Gammaproteobacteria; Legionellales; Legionellaceae; *Legionella*	0.016	0.111	0
Proteobacteria; Gammaproteobacteria; Xanthomonadales; Sinobacteraceae; *Alkanibacter*	0.304	0.142	0
Proteobacteria; Gammaproteobacteria; Xanthomonadales; Sinobacteraceae; *Steroidobacter*	0.135	0.190	0
Proteobacteria; Gammaproteobacteria; Xanthomonadales; Xanthomonadaceae; *Dokdonella*	0.033	0.047	0
Proteobacteria; Gammaproteobacteria; Xanthomonadales; Xanthomonadaceae; *Lysobacter*	0.270	0.222	0
Proteobacteria; Gammaproteobacteria; Xanthomonadales; Xanthomonadaceae; *Rhodanobacter*	0.879	0.301	0
Proteobacteria; Gammaproteobacteria; Xanthomonadales; Xanthomonadaceae; *Thermomonas*	0.169	0.349	0
Verrucomicrobia; Opitutae; Opitutales; Opitutaceae; *Opitutus*	0.033	0.111	0
Verrucomicrobia; Verrucomicrobiae; Verrucomicrobiales; Verrucomicrobiaceae; *Luteolibacter*	0.101	0.047	0

Table S3 Relative abundance of bacterial families found in the substrate, and associated with the rhizosphere, roots, stem, leaves, spathe and spadix tissues of healthy *Anthurium andraeanum* L. plants.

Taxon	Substrate	Rhizos-phere	Root	Stem	Leave	Spathe	Spadix
	Relative abundance (%)						
Other Bacteria	11.01 (0.62)[a]	11.10 (1.26)	11.93 (4.33)	15.69 (6.79)	27.74 (12.80)	16.49 (4.58)	23.52 (9.93)
**Acidobacteria**							
Acidobacteria	0.07 (0.07)	0	0	0	0	0	0
Acidobacteria-5	0.25 (0.11)	0.07 (0.03)	0	0	0	0	0
Acidobacteria-6	0.13 (0.13)	0	0	0	0	0	0
Acidobacteria-6; BPC015	0.16 (0.09)	0.35 (0.27)	0	0	0	0	0
Acidobacteria-6; CCU21	0.35 (0.07)	0.18 (0.09)	0	0	0	0	0
Acidobacteria-6; iii1-15	0.03 (0.02)	0.02 (0.01)	0	0	0	0	0
Acidobacteria-6; iii1-15	4.83 (1.01)	3.20 (0.41)	0	0	0	0	0
Acidobacteria-6; iii1-15; RB40	0.89 (0.30)	.04 (0.40)	0	0	0	0	0
Acidobacteria-6; iii1-15; mb2424	0.59 (0.12)	0.40 (0.18)	0	0	0	0	0
Acidobacteriia; Acidobacteriales	0.05 (0.03)	0.01 (0.01)	0	0	0	0	0
Acidobacteriia; Acidobacteriales; Acidobacteriaceae	4.10 (2.02)	4.37 (1.58)	0.22 (0.12)	0	0	0	0
Acidobacteriia; Acidobacteriales; Koribacteraceae	0.29 (0.10)	0.22 (0.13)	0	0	0	0	0
BPC102; MVS-40	0.03 (0.03)	0.02 (0.02)	0	0	0	0	0
DA052; Ellin6513	0.02 (0.02)	0.07 (0.03)	0	0	0	0	0
EC1113	0	0.12 (0.06)	0	0	0	0	0
PAUC37f	0.03 (0.03)	0.02 (0.02)	0	0	0	0	0
RB25	0.11 (0.08)	0.03 (0.03)	0	0	0	0	0
S035	0.03 (0.03)	0.02 (0.02)	0	0	0	0	0
Solibacteres; Solibacterales	0.11 (0.03)	0.20 (0.07)	0	0	0	0	0
Solibacteres; Solibacterales; PAUC26f	0.03 (0.03)	0.05 (0.05)	0	0	0	0	0
Solibacteres; Solibacterales; Solibacteraceae	0.03 (0.02)	0.03 (0.03)	0	0	0	0	0
Sva0725; Sva0725	0.10 (0.07)	0.20 (0.08)	0	0	0	0	0
[Chloracidobacteria]; RB41	0.13 (0.04)	0.06 (0.03)	0	0	0	0	0
[Chloracidobacteria]; RB41; Ellin6075	2.05 (0.45)	2.23 (0.91)	0	0	0	0	0
iii1-8; 32-20	0.05 (0.03)	0	0	0	0	0	0
iii1-8; DS-18	0	0.12 (0.12)	0	0	0	0	0
**Actinobacteria**							
Acidimicrobiia; Acidimicrobiales	0.89 (0.47)	1.50 (0.89)	0	0	0	0	0
Acidimicrobiia; Acidimicrobiales; C111	0.16 (0.11)	0.08 (0.05)	0	0	0	0	0
Acidimicrobiia; Acidimicrobiales; EB1017	0.34 (0.12)	0.31 (0.11)	0	0	0	0	0
Acidimicrobiia; Acidimicrobiales; koll13	0.03 (0.03)	0.01 (0.01)	0	0	0	0	0
Actinobacteria; Actinomycetales	0.68 (0.19)	0.42 (0.07)	0.52 (0.42)	0	0	0	0
Actinobacteria; Actinomycetales; Cellulomonadaceae	0	0.02 (0.02)	0	0	0	0	0
Actinobacteria; Actinomycetales; Microbacteriaceae	0.54 (0.06)	0.49 (0.10)	1.82 (1.51)	0	0	0	0
Actinobacteria; Actinomycetales; Micromonosporaceae	0.08 (0.04)	0.18 (0.09)	0.77 (0.65)	0	0	0	0
Actinobacteria; Actinomycetales; Mycobacteriaceae	0.43 (0.14)	0.30 (0.12)	0	0	0	0	0
Actinobacteria; Actinomycetales; Nakamurellaceae	0.06 (0.04)	0	0	0	0	0	0
Actinobacteria; Actinomycetales; Nocardioidaceae	0.38 (0.25)	0.27 (0.11)	0	0	0	0	0
Actinobacteria; Actinomycetales; Nocardiopsaceae	0	0.02 (0.02)	0	0	0	0	0
Actinobacteria; Actinomycetales; Promicromonosporaceae	0.03 (0.03)	0.01 (0.01)	0	0	0	0	0
Actinobacteria; Actinomycetales; Propionibacteriaceae	0.02 (0.02)	0.15 (0.14)	0.65 (0.45)	1.05 (0.65)	0	0.48 (0.28)	0
Actinobacteria; Actinomycetales; Pseudonocardiaceae	0.07 (0.06)	0.02 (0.02)	0	0	0	0	0
Actinobacteria; Actinomycetales; Streptomycetaceae	0.07 (0.06)	0.28 (0.19)	3.22 (2.20)	0	0	0	0
Actinobacteria; Actinomycetales; Williamsiaceae	0.02 (0.02)	0.02 (0.02)	0	0	0	0	0
MB-A2-108	0	0.02 (0.02)	0	0	0	0	0
MB-A2-108; 0319-7L14;	0.04 (0.03)	0.11 (0.07)	0	0	0	0	0
Thermoleophilia; Gaiellales; Gaiellaceae	1.33 (0.62)	1.65 (0.67)	0	0	0	0	0
Thermoleophilia; Solirubrobacterales	0.30 (0.11)	0.27 (0.11)	0	0	0	0	0
Thermoleophilia; Solirubrobacterales; Conexibacteraceae	0.13 (0.06)	0.12 (0.07)	0	0	0	0	0
Thermoleophilia; Solirubrobacterales; Patulibacteraceae	0.04 (0.03)	0.02 (0.02)	0	0	0	0	0
Thermoleophilia; Solirubrobacterales; Solirubrobacteraceae	0	0.04 (0.03)	0	0	0	0	0
**Bacteroidetes**							
Cytophagia; Cytophagales; Cytophagaceae	0.93 (0.38)	1.26 (0.14)	3.10 (1.90)	0	0	0	0
Flavobacteriia; Flavobacteriales	0.02 (0.02)	0	0	0	0	0	0
Flavobacteriia; Flavobacteriales; Cryomorphaceae	0.04 (0.03)	0.01 (0.01)	0	0	0	0	0
Flavobacteriia; Flavobacteriales; Flavobacteriaceae	0.39 (0.15)	0.36 (0.18)	4.36 (2.84)	0	0	0	0
Sphingobacteriia; Sphingobacteriales;	0.25 (0.15)	0.03 (0.03)	0	0	0	0	0

Table S3 (continued)

Taxon	Substrate	Rhizos-phere	Root	Stem	Leave	Spathe	Spadix
Sphingobacteriia; Sphingobacteriales; Sphingobacteriaceae	0.05 (0.05)	0	0	0	0	0	0
[Saprospirae]; [Saprospirales]; Chitinophagaceae	1.59 (0.33)	1.46 (0.44)	0	0	0	0	0
[Saprospirae]; [Saprospirales]; Saprospiraceae	0.05 (0.03)	0.04 (0.04)	0	0	0	0	0
**Chloroflexi**							
Chloroflexi	0.02 (0.02)	0.14 (0.07)	0	0	0	0	0
Anaerolineae; A31; S47	0.04 (0.03)	0.05 (0.04)	0	0	0	0	0
Anaerolineae; CFB-26	0.04 (0.03)	0	0	0	0	0	0
Anaerolineae; Caldilineales; Caldilineaceae	0.15 (0.15)	0.11 (0.11)	0	0	0	0	0
Anaerolineae; DRC31	0.01 (0.01)	0.29 (0.18)	0	0	0	0	0
Anaerolineae; GCA004	0.03 (0.03)	0.14 (0.08)	0	0	0	0	0
Anaerolineae; H39	0.08 (0.05)	0	0	0	0	0	0
Anaerolineae; S0208	0.20 (0.14)	0.10 (0.04)	0	0	0	0	0
Anaerolineae; SBR1031; A4b	0.26 (0.09)	0.17 (0.08)	0	0	0	0	0
Anaerolineae; envOPS12	0.04 (0.03)	0.09 (0.03)	0	0	0	0	0
Chloroflexi; [Roseiflexales]	0.01 (0.01)	0.06 (0.04)	0	0	0	0	0
Ellin6529	0.85 (0.40)	1.41 (0.35)	0	0	0	0	0
Gitt-GS-136	0.55 (0.24)	0.36 (0.14)	0	0	0	0	0
Ktedonobacteria	0	0.06 (0.06)	0	0	0	0	0
Ktedonobacteria; JG30-KF-AS9	0.03 (0.03)	0.06 (0.06)	0	0	0	0	0
S085	1.51 (0.31)	1.33 (0.42)	0	0	0	0	0
SAR202	0	0.10 (0.10)	0	0	0	0	0
TK10	0	0.05 (0.03)	0	0	0	0	0
TK10; AKYG885; 5B-12	0.09 (0.06)	0.07 (0.07)	0	0	0	0	0
TK10; B07_WMSP1	0.03 (0.03)	0.02 (0.01)	0	0	0	0	0
TK10; B07_WMSP1	0.05 (0.03)	0.06 (0.06)	0	0	0	0	0
TK17	0.41 (0.17)	0.22 (0.00)	0	0	0	0	0
TK17; mle1-48	0.54 (0.19)	0.85 (0.30)	0	0	0	0	0
Thermomicrobia; AKYG1722	0.06 (0.06)	0.03 (0.02)	0	0	0	0	0
**Firmicutes**							
Bacilli; Bacillales	0.11 (0.06)	0.28 (0.03)	0	0	0	0	0
Bacilli; Bacillales; Alicyclobacillaceae	0.09 (0.09)	0.01 (0.01)	0	0	0	0	0.77 (0.57)
Bacilli; Bacillales; Bacillaceae	1.36 (0.75)	1.07 (0.42)	0	0	0	0	0
Bacilli; Bacillales; Paenibacillaceae	0.20 (0.10)	0.15 (0.06)	2.77 (0.91)	2.05 (1.26)	6.25 (4.84)	10.17 (3.90)	11.73 (3.66)
Bacilli; Bacillales; Planococcaceae	0.26 (0.15)	0.21 (0.10)	0	0	0	0	0
Bacilli; Bacillales; Thermoactinomycetaceae	0.51 (0.25)	0.27 (0.12)	0	0	0	0	0
Bacilli; Haloplasmatales; Haloplasmataceae	0	0	0	0	0	1.90 (1.90)	0
Bacilli; Lactobacillales; Aerococcaceae	0	0	0.23 (0.23)	0	5.00 (3.00)	1.68 (1.18)	2.93 (1.59)
Clostridia	0.02 (0.02)	0	0	0	0	0	0
Clostridia; Clostridiales	0	0	0	0	0	0	0.83 (0.36)
Clostridia; Clostridiales; Clostridiaceae	0.23 (0.14)	0.07 (0.07)	0.77 (0.57)	4.72 (2.64)	5.80 (4.40)	1.69 (1.25)	3.16 (1.64)
Clostridia; Clostridiales; Lachnospiraceae	0	0	4.51 (2.03)	6.13 (3.67)	2.78 (1.87)	17.12 (5.23)	8.62 (3.16)
Clostridia; Clostridiales; Peptostreptococcaceae	0.02 (0.02)	0.16 (0.11)	3.90 (3.02)	10.00 (3.10)	0	0.48 (0.28)	9.37 (5.67)
Clostridia; Clostridiales; Veillonellaceae	0	0	1.20 (0.78)	4.21 (2.78)	0	4.55 (1.67)	3.62 (1.63)
**Planctomycetes**							
Phycisphaerae; CCM11a	0	0.06 (0.06)	0	0	0	0	0
Phycisphaerae; Cpla-3	0.42 (0.22)	0.41 (0.16)	0	0	0	0	0
Phycisphaerae; Phycisphaerales	0.20 (0.16)	0.10 (0.10)	0	0	0	0	0
Phycisphaerae; Pla1	0.08 (0.08)	0.03 (0.02)	0	0	0	0	0
Phycisphaerae; S-70	0.07 (0.07)	0.02 (0.02)	0	0	0	0	0
Phycisphaerae; WD2101	0.53 (0.16)	0.27 (0.03)	0	0	0	0	0
Planctomycetia; Gemmatales; Isosphaeraceae	0	0.10 (0.10)	0	0	0	0	0
Planctomycetia; Pirellulales; Pirellulaceae	0.29 (0.10)	0.21 (0.09)	0	0	0	0	0
Planctomycetia; Planctomycetales; Planctomycetaceae	0	0.10 (0.10)	0	0	0	0	0
vadinHA49; DH61	0.05 (0.05)	0	0	0	0	0	0
**Alphaproteobacteria**							
Proteobacteria	0.30 (0.19)	0.32 (0.17)	0.43 (0.43)	0	0.63 (0.31)	0.65 (0.56)	0
Proteobacteria; Alphaproteobacteria	1.92 (0.37)	2.24 (0.21)	0.47 (0.35)	0	0	0	0
BD7-3	0.03 (0.03)	0.04 (0.03)	0	0	0	0	0
Caulobacterales; Caulobacteraceae	0.90 (0.29)	1.34 (0.46)	1.18 (1.18)	0	0	0.95 (0.48)	0
Ellin329	0.11 (0.03)	0.13 (0.08)	0	0	0	0	0
Kiloniellales	0.02 (0.02)	0.02 (0.02)	0	0	0	0	0

Table S3 (continued)

Taxon	Substrate	Rhizos-phere	Root	Stem	Leave	Spathe	Spadix
Rhizobiales	3.19 (0.48)	2.92 (0.55)	0	0	0	0	0
Rhizobiales; Beijerinckiaceae	0.07 (0.07)	0.07 (0.05)	0	0	0	0	0
Rhizobiales; Bradyrhizobiaceae	1.06 (0.22)	0.52 (0.07)	0.49 (0.30)	0	0	0	0
Rhizobiales; Hyphomicrobiaceae	10.38 (2.08)	12.32 (2.39)	1.68 (0.75)	0	0	0	0
Rhizobiales; Methylobacteriaceae	0.21 (0.04)	0.16 (0.06)	0	0	0	0	0
Rhizobiales; Methylocystaceae	0.19 (0.09)	0.07 (0.03)	0	0	0	0	0
Rhizobiales; Phyllobacteriaceae	0.57 (0.17)	0.55 (0.14)	0	0	0	0	0
Rhizobiales; Rhizobiaceae	0.06 (0.03)	0.49 (0.12)	0.88 (0.39)	0	0	2.61 (1.43)	0
Rhizobiales; Rhodobiaceae	0.08 (0.05)	0.04 (0.03)	0	0	0	0	0
Rhizobiales; Xanthobacteraceae	0.07 (0.07)	0.07 (0.07)	0	0	0	0	0
Rhodobacterales; Hyphomonadaceae	0.19 (0.07)	0.24 (0.17)	0	0	0	0	0
Rhodobacterales; Rhodobacteraceae	0.05 (0.03)	0.23 (0.20)	0	0	0	0	0
Rhodospirillales	0.10 (0.07)	0.04 (0.02)	0	0	0	0	0
Rhodospirillales; Acetobacteraceae	0.46 (0.20)	0.57 (0.30)	0	0.48 (0.48)	0	0	0
Rhodospirillales; Rhodospirillaceae	7.31 (1.38)	6.36 (0.85)	0.95 (0.59)	0	0	0	0
Sphingomonadales	0.39 (0.11)	0.16 (0.08)	0	0	0	0	0
Sphingomonadales; Erythrobacteraceae	0.47 (0.11)	0.52 (0.26)	0	0	0	0	0
Sphingomonadales; Sphingomonadaceae	0.49 (0.25)	0.51 (0.11)	0	0	0	0	0
**Betaproteobacteria**							
Proteobacteria; Betaproteobacteria	1.41 (0.31)	1.24 (0.42)	0	0	0	0	0
Burkholderiales	0.03 (0.03)	0.14 (0.10)	0	0	0	0	0
Burkholderiales; Alcaligenaceae	0.15 (0.07)	0.15 (0.13)	9.15 (6.67)	2.92 (1.26)	0	0.40 (0.20)	1.96 (1.49)
Burkholderiales; Burkholderiaceae	0.66 (0.21)	1.10 (0.32)	0	0	0	0	0
Burkholderiales; Comamonadaceae	0.94 (0.47)	0.81 (0.23)	0.39 (0.17)	3.81 (1.82)	0.63 (0.33)	0	0.42 (0.22)
Burkholderiales; Oxalobacteraceae	0.15 (0.07)	0.15 (0.09)	0.52 (0.21)	2.38 (1.82)	0	0	0
Ellin6067	0.11 (0.04)	0.03 (0.02)	0	0	0	0	0
IS-44	0.07 (0.07)	0.03 (0.03)	0	0	0	0	0
MND1	0.44 (0.17)	0.44 (0.20)	0	0	0	0	0
Nitrosomonadales; Nitrosomonadaceae	0.10 (0.04)	0.07 (0.06)	0	0	0	0	0
Rhodocyclales; Rhodocyclaceae	0.08 (0.08)	0.02 (0.02)	0.65 (0.45)	0	0	0.40 (0.20)	0
SC-I-84	0.47 (0.12)	0.56 (0.25)	0	0	0	0	0
**Deltaproteobacteria**							
Proteobacteria; Deltaproteobacteria	0.01 (0.01)	0.16 (0.08)	0	0	0	0	0
Bdellovibrionales; Bdellovibrionaceae	0	0.02 (0.02)	0	0	0	0	0
DTB120	0.03 (0.03)	0	0	0	0	0	0
MIZ46	0.09 (0.05)	0.08 (0.04)	0	0	0	0	0
Myxococcales	0.16 (0.04)	0.27 (0.15)	0	0	0	0	0
Myxococcales; Haliangiaceae	0.10 (0.05)	0.04 (0.02)	0	0	0	0	0
Myxococcales; Nannocystaceae	0	0.15 (0.10)	0	0	0	0	0
Myxococcales; OM27	0.17 (0.09)	0.05 (0.03)	0	0	0	0	0
Myxococcales; Polyangiaceae	0.05 (0.05)	0.02 (0.02)	0	0	0	0	0
NB1-j; MND4	0.09 (0.03)	0.08 (0.03)	0	0	0	0	0
NB1-j; NB1-i	0.03 (0.03)	0.02 (0.02)	0	0	0	0	0
Spirobacillales	0	0.02 (0.02)	0	0	0	0	0
Syntrophobacterales; Syntrophobacteraceae	1.12 (0.32)	1.91 (0.32)	0	0	0	0	0
**Gammaproteobacteria**							
Proteobacteria; Gammaproteobacteria	0.51 (0.18)	0.38 (0.16)	0	0	0	0	0
Aeromonadales; Aeromonadaceae	0.09 (0.09)	0.01 (0.01)	0	0	0	0	0
Alteromonadales	0.35 (0.20)	0.23 (0.10)	0	0	0	0	0
Alteromonadales; 125ds10	0.13 (0.10)	0.11 (0.11)	0	0	0	0	0
Alteromonadales; OM60	0.33 (0.24)	0.30 (0.10)	0	0	0	0	0
Chromatiales	0.01 (0.01)	0.01 (0.01)	0	0	0	0	0
Enterobacteriales; Enterobacteriaceae	0.03 (0.02)	0.01 (0.01)	5.71 (2.91)	0	0	0	0.42 (0.22)
HTCC2188; HTCC2089	0.10 (0.05)	0.35 (0.08)	0	0	0	0	0
Legionellales	0.11 (0.03)	0.22 (0.06)	0	0	0	0	0
Legionellales; Coxiellaceae	0.36 (0.12)	0.28 (0.06)	0	0	0	0	0
Legionellales; Legionellaceae	0.03 (0.03)	0.08 (0.07)	0	0	0	0	0
Oceanospirillales; Halomonadaceae	0.39 (0.09)	0.41 (0.15)	0.68 (0.36)	0	0.63 (0.43)	1.12 (0.70)	0
Pseudomonadales; Moraxellaceae	0.50 (0.37)	0.04 (0.04)	9.05 (4.79)	0.48 (0.48)	7.50 (3.11)	4.45 (3.70)	0
Pseudomonadales; Pseudomonadaceae	0.50 (0.03)	0.40 (0.21)	15.70 (6.42)	44.01 (9.37)	40.33 (10.76)	30.56 (5.35)	31.85 (4.94)
Thiotrichales; Piscirickettsiaceae	0.47 (0.07)	0.39 (0.22)	0	0	0	0	0
Xanthomonadales; Sinobacteraceae	2.21 (0.53)	1.91 (0.34)	0.77 (0.57)	0	0	0	0
Xanthomonadales; Xanthomonadaceae	4.43 (1.95)	3.64 (1.15)	4.76 (2.21)	2.07 (0.97)	2.73 (1.68)	0	0.80 (0.49)
[Marinicellales]; [Marinicellaceae]	0.08 (0.08)	0	0	0	0	0	0

Table S3 (continued)

Taxon	Substrate	Rhizos-phere	Root	Stem	Leave	Spathe	Spadix
**Other Phyla**							
Armatimonadetes; 0319-6E2	0.02 (0.02)	0	0	0	0	0	0
Armatimonadetes; [Fimbriimonadia]; [Fimbriimonadales]	0.01 (0.01)	0.01 (0.01)	0	0	0	0	0
Armatimonadetes; [Fimbriimonadia]; [Fimbriimonadales]	0	0.19 (0.07)	0	0	0	0	0
Armatimonadetes; [Fimbriimonadia]; [Fimbriimonadales]; [Fimbriimonadaceae]	0.85 (0.22)	0.75 (0.35)	0	0	0	2.40 (1.40)	0
BRC1; NPL-UPA2	0.02 (0.02)	0.02 (0.02)	0	0	0	0	0
BRC1; PRR-11	0.03 (0.03)	0	0	0	0	0	0
Chlorobi	0.04 (0.03)	0.07 (0.07)	0	0	0	0	0
Chlorobi; Ignavibacteria; Ignavibacteriales; Ignavibacteriaceae	0.19 (0.09)	0.29 (0.15)	0	0	0	0	0
Chlorobi; OPB56	0.01 (0.01)	0.08 (0.07)	0	0	0	0	0
Chlorobi; SJA-28	0.12 (0.04)	0.17 (0.11)	0	0	0	0	0
Cyanobacteria	0	0	0	0	0	1.90 (1.00)	0
Cyanobacteria; 4C0d-2; MLE1-12	0.04 (0.04)	0.06 (0.06)	0	0	0	0	0
Cyanobacteria; 4C0d-2; SM1D11	0	0.08 (0.06)	0	0	0	0	0
Elusimicrobia; Elusimicrobia; IIb	0	0.03 (0.03)	0	0	0	0	0
GN02; GKS2-174	0	0.02 (0.02)	0	0	0	0	0
GN04; GN15	0.04 (0.04)	0.05 (0.03)	0	0	0	0	0
GOUTA4	0.05 (0.03)	0	0	0	0	0	0
Gemmatimonadetes; Gemm-1	0.83 (0.28)	1.20 (0.35)	0	0	0	0	0
Gemmatimonadetes; Gemm-2	0.01 (0.01)	0.03 (0.02)	0	0	0	0	0
Gemmatimonadetes; Gemm-3	0	0.05 (0.03)	0	0	0	0	0
Gemmatimonadetes; Gemm-5	0.11 (0.07)	0.03 (0.02)	0	0	0	0	0
Gemmatimonadetes; Gemmatimonadetes	0.55 (0.19)	0.25 (0.14)	0	0	0	0	0
Gemmatimonadetes; Gemmatimonadetes; Ellin5290	0.03 (0.02)	0.07 (0.07)	0	0	0	0	0
Gemmatimonadetes; Gemmatimonadetes; N1423WL	0.10 (0.07)	0.13 (0.07)	0	0	0	0	0
Kazan-3B-28	0.03 (0.03)	0	0	0	0	0	0
MVP-21	0.14 (0.06)	0.24 (0.12)	0	0	0	0	0
NKB19	0.03 (0.03)	0.18 (0.13)	0	0	0	0	0
Nitrospirae; Nitrospira; Nitrospirales; 0319-6A21	0.11 (0.08)	0.05 (0.05)	0	0	0	0	0
Nitrospirae; Nitrospira; Nitrospirales; Nitrospiraceae	1.34 (0.44)	0.96 (0.25)	0	0	0	0	0
OD1	0.03 (0.03)	0	0	0	0	0	0
OD1; ABY1	0.27 (0.20)	0.06 (0.06)	0	0	0	0	0
OD1; SM2F11	0.10 (0.05)	0.14 (0.06)	0	0	0	0	0
OD1; ZB2	0.07 (0.05)	0.04 (0.03)	0	0	0	0	0
OP11	0.24 (0.07)	0.15 (0.10)	0	0	0	0	0
OP11; OP11-4	0.03 (0.03)	0.04 (0.03)	0	0	0	0	0
OP11; WCHB1-64	0.02 (0.02)	0	0	0	0	0	0
SBR1093; VHS-B5-50	0.21 (0.09)	0.04 (0.03)	0	0	0	0	0
TM6; SJA-4	0.34 (0.11)	0.24 (0.07)	0	0	0	0	0
TM6; SJA-4; YJF2-48	0.02 (0.02)	0.04 (0.04)	0	0	0	0	0
TM7	0.26 (0.20)	0.34 (0.34)	0	0	0	0	0
TM7; TM7-1	3.34 (0.76)	3.52 (0.62)	0	0	0	0	0
TM7; TM7-3	0.03 (0.03)	0.07 (0.07)	0	0	0	0	0
TM7; TM7-3	0	0	1.04 (0.56)	0	0	0	0
Tenericutes; Mollicutes; Anaeroplasmatales; Anaeroplasmataceae	0	0	4.13 (3.15)	0	0	0	0
Verrucomicrobia; Opitutae; Opitutales; Opitutaceae	0.03 (0.02)	0.12 (0.06)	0	0	0	0	0
Verrucomicrobia; Verrucomicrobiae; Verrucomicrobiales; Verrucomicrobiaceae	0.09 (0.09)	0.12 (0.05)	0	0	0	0	0
Verrucomicrobia; [Pedosphaerae]; [Pedosphaerales]	0.03 (0.02)	0.08 (0.06)	0	0	0	0	0
Verrucomicrobia; [Pedosphaerae]; [Pedosphaerales]; auto67_4W	0.17 (0.12)	0.04 (0.03)	0	0	0	0	0
Verrucomicrobia; [Spartobacteria]; [Chthoniobacterales]; [Chthoniobacteraceae]	0.03 (0.03)	0.06 (0.06)	0	0	0	0	0
WPS-2	0.17 (0.08)	0.11 (0.06)	0	0	0	0	0
WS2; SHA-109	0.04 (0.03)	0.05 (0.03)	0	0	0	0	0
WS3; PRR-12; GN03; KSB4	0.03 (0.03)	0.01 (0.01)	0	0	0	0	0
WS3; PRR-12; LD1-PA13	0.06 (0.04)	0.22 (0.10)	0	0	0	0	0
WS3; PRR-12; Sediment-1	0.11 (0.08)	0.40 (0.17)	0	0	0	0	0
WS3; PRR-12; Sediment-1; CV106	0	0.02 (0.02)	0	0	0	0	0

[a] Numbers in parenthesis are the standard errors (*n* = 5)

Table S4 Relative abundance of bacterial genera found in the substrate, and associated with the rhizosphere, roots, stem, leaves, spathe and spadix tissues of healthy *Anthurium andraeanum* L. plants.

Taxon	Substrate	Rhizos-phere	Root	Stem	Leaves	Spathe	Spadix
	Relative abundance (%)						
**Acidobacteria**							
Solibacteres; Solibacterales; Solibacteraceae; Candidatus *Solibacter*	0.02 (0.02)[a]	0	0	0	0	0	0
**Actinobacteria**							
Actinomycetales; Cellulomonadaceae; *Demequina*	0	0.02 (0.02)	0	0	0	0	0
Actinomycetales; Microbacteriaceae; *Agromyces*	0.11 (0.08)	0.04 (0.02)	0	0	0	0	0
Actinomycetales; Microbacteriaceae; *Cryocola*	0.08 (0.04)	0.01 (0.01)	0	0	0	0	0
Actinomycetales; Microbacteriaceae; *Microbacterium*	0.06 (0.03)	0.09 (0.07)	1.82 (1.51)	0	0	0	0
Actinomycetales; Mycobacteriaceae; *Mycobacterium*	0.43 (0.14)	0.30 (0.12)	0	0	0	0	0
Actinomycetales; Nocardioidaceae; *Nocardioides*	0.33 (0.22)	0.25 (0.11)	0	0	0	0	0
Actinomycetales; Nocardiopsaceae; *Thermobifida*	0	0.02 (0.02)	0	0	0	0	0
Actinomycetales; Promicromonosporaceae; *Xylanimicrobium*	0.03 (0.03)	0.01 (0.01)	0	0	0	0	0
Actinomycetales; Propionibacteriaceae; *Propionibacterium*	0	0.14 (0.14)	0.65 (0.45)	1.05 (0.65)	0	0.48 (0.28)	0
Actinomycetales; Pseudonocardiaceae; *Saccharomonospora*	0.01 (0.01)	0.02 (0.02)	0	0	0	0	0
Actinomycetales; Streptomycetaceae; *Streptomyces*	0	0.28 (0.19)	3.22 (2.20)	0		0	0
Actinomycetales; Williamsiaceae; *Williamsia*	0.02 (0.02)	0.02 (0.02)	0	0	0	0	0
Thermoleophilia; Solirubrobacterales; Conexibacteraceae; *Conexibacter*	0.13 (0.06)	0.12 (0.07)	0	0	0	0	0
**Bacteroidetes**							
Cytophagia; Cytophagales; Cytophagaceae; *Adhaeribacter*	0.08 (0.07)	0.18 (0.14)	0	0	0	0	0
Flavobacteriia; Flavobacteriales; Flavobacteriaceae; *Flavobacterium*	0.39 (0.15	0.36 (0.18)	4.36 (2.84)	0	0	0	0
Sphingobacteriia; Sphingobacteriales; Sphingobacteriaceae; *Solitalea*	0.05 (0.05)	0	0	0	0	0	0
[Saprospirae]; [Saprospirales]; Chitinophagaceae; *Flavihumibacter*	0.03 (0.03)	0.02 (0.02)	0	0	0	0	0
[Saprospirae]; [Saprospirales]; Chitinophagaceae; *Flavisolibacter*	0	0.02 (0.02)	0	0	0	0	0
**Chloroflexi**							
Anaerolineae; Caldilineales; Caldilineaceae; *Caldilinea*	0	0.02 (0.02)	0	0	0	0	0
**Firmicutes**							
Bacilli; Bacillales; Alicyclobacillaceae; *Alicyclobacillus*	0.09 (0.09)	0.01 (0.01)	0	0	0	0	0.77 (0.57)
Bacilli; Bacillales; Bacillaceae; *Bacillus*	1.11 (0.61)	0.78 (0.29)	0	0	0	0	0
Bacilli; Bacillales; Bacillaceae; *Ornithinibacillus*	0.02 (0.02)	0	0	0	0	0	0
Bacilli; Bacillales; Paenibacillaceae; *Brevibacillus*	0.07 (0.07)	0.06 (0.04)	2.77 (0.91)	2.05 (1.26)	6.25 (4.84)	10.17 (3.90)	11.73 (3.66)
Bacilli; Bacillales; Paenibacillaceae; *Paenibacillus*	0.06 (0.05)	0.03 (0.03)	0	0	0	0	0
Bacilli; Bacillales; Paenibacillaceae; *Thermobacillus*	0	0.05 (0.05)	0	0	0	0	0
Bacilli; Bacillales; Planococcaceae; *Lysinibacillus*	0.03 (0.02)	0.02 (0.02)	0	0	0	0	0
Bacilli; Bacillales; Planococcaceae; *Solibacillus*	0.10 (0.06)	0.12 (0.06)	0	0	0	0	0
Bacilli; Bacillales; Planococcaceae; *Ureibacillus*	0.11 (0.09)	0	0	0	0	0	0
Bacilli; Bacillales; Thermoactinomycetaceae;	0.09 (0.06)	0	0	0	0	0	0
Bacilli; Bacillales; Thermoactinomycetaceae; *Laceyella*	0	0.02 (0.02)	0	0	0	0	0
Bacilli; Bacillales; Thermoactinomycetaceae; *Planifilum*	0.41 (0.20)	0.25 (0.13)	0	0	0	0	0
Bacilli; Bacillales; Thermoactinomycetaceae; *Thermoactinomyces*	0.02 (0.02)	0	0	0	0	0	0
Bacilli; Lactobacillales; Aerococcaceae; *Alkalibacterium*	0	0	0.23 (0.23)	0	5.00 (3.00)	1.68 (1.18)	2.93 (1.59)
Clostridia; Clostridiales; Clostridiaceae; *Alkaliphilus*	0	0	0	0.69 (0.69)	0.63 (0.63)	0.65 (0.65)	0.42 (0.42)
Clostridia; Clostridiales; Clostridiaceae; *Clostridium*	0.04 (0.03)	0.01 (0.01)	0.77 (0.57)	2.86 (1.66)	4.26 (3.53)	1.05 (0.67)	2.75 (1.34)
Clostridia; Clostridiales; Clostridiaceae; *Oxobacter*	0	0.04 (0.04)	0	0	0	0	0
Clostridia; Clostridiales; Peptostreptococcaceae; *Clostridium*	0	0.03 (0.03)	3.90 (3.02)	6.00 (2.08)	0	0.48 (0.28)	1.37 (0.86)
Clostridia; Clostridiales; Peptostreptococcaceae; [*Clostridium*]	0.02 (0.02)	0.08 (0.05)	0	4.00 (1.02)	0	0	8.00 (8.00)
Clostridia; Clostridiales; Veillonellaceae; *Pelosinus*	0	0	1.20 (0.78)	4.21 (2.78)	0	4.55 (1.67)	3.62 (1.63)
**Planctomycetes**							
Planctomycetia; Planctomycetales; Planctomycetaceae; *Planctomyces*	0	0.10 (0.10)	0	0	0	0	0
**Alphaproteobacteria**							
Caulobacterales; Caulobacteraceae; *Asticcacaulis*	0	0.02 (0.02)	0	0	0	0	0
Caulobacterales; Caulobacteraceae; *Phenylobacterium*	0.41 (0.21)	0.59 (0.34)	0	0	0	0	0
Rhizobiales; Bradyrhizobiaceae; *Balneimonas*	0.09 (0.06)	0.18 (0.07)	0	0	0	0	0
Rhizobiales; Bradyrhizobiaceae; *Bradyrhizobium*	0.34 (0.10)	0.13 (0.03)	0.49 (0.30)	0	0	0	0
Rhizobiales; Hyphomicrobiaceae; *Devosia*	0.94 (0.32)	1.64 (0.48)	0.91 (0.57)	0	0	0	0

Table S4 (continued)

**Taxon**	**Substrate**	**Rhizos-phere**	**Root**	**Stem**	**Leaves**	**Spathe**	**Spadix**
Rhizobiales; Hyphomicrobiaceae; *Hyphomicrobium*	0.44 (0.12)	1.13 (0.61)	0	0	0	0	0
Rhizobiales; Hyphomicrobiaceae; *Pedomicrobium*	0.58 (0.22)	0.41 (0.15)	0	0	0	0	0
Rhizobiales; Hyphomicrobiaceae; *Rhodoplanes*	8.05 (1.79)	8.43 (1.44)	0	0	0	0	0
Rhizobiales; Methylobacteriaceae; *Methylobacterium*	0.21 (0.04)	0.16 (0.06)	0	0	0	0	0
Rhizobiales; Methylocystaceae; *Pleomorphomonas*	0	0.02 (0.02)	0	0	0	0	0
Rhizobiales; Phyllobacteriaceae; *Mesorhizobium*	0.41 (0.12)	0.34 (0.06)	0	0	0	0	0
Rhizobiales; Rhizobiaceae; *Agrobacterium*	0.03 (0.02)	0.05 (0.04)	0	0	0	0	0
Rhizobiales; Rhizobiaceae; *Rhizobium*	0.01 (0.01)	0	0	0	0	2.61 (1.43)	0
Rhizobiales; Rhizobiaceae; *Sinorhizobium*	0.02 (0.02)	0.43 (0.09)	0.88 (0.39)	0	0	0	0
Rhizobiales; Rhodobiaceae; *Afifella*	0.08 (0.05)	0.04 (0.03)	0	0	0	0	0
Rhizobiales; Xanthobacteraceae; *Labrys*	0.07 (0.07)	0.07 (0.07)	0	0	0	0	0
Rhodobacterales; Rhodobacteraceae; *Amaricoccus*	0.05 (0.03)	0.13 (0.10)	0	0	0	0	0
Sphingomonadales; Erythrobacteraceae; *Altererythrobacter*	0.02 (0.02)	0	0	0	0	0	0
Sphingomonadales; Sphingomonadaceae; *Novosphingobium*	0	0.04 (0.04)	0	0	0	0	0
Sphingomonadales; Sphingomonadaceae; *Sphingobium*	0.10 (0.05)	0.18 (0.07)	0	0	0	0	0
Sphingomonadales; Sphingomonadaceae; *Sphingopyxis*	0.03 (0.02)	0.04 (0.03)	0	0	0	0	0
**Betaproteobacteria**							
Burkholderiales; Alcaligenaceae; *Achromobacter*	0	0.02 (0.02)	6.06 (4.29)	2.92 (1.26)	0	0.40 (0.20)	1.96 (1.49)
Burkholderiales; Burkholderiaceae; *Burkholderia*	0.04 (0.03)	0.04 (0.02)	0	0	0	0	0
Burkholderiales; Comamonadaceae; *Acidovorax*	0.01 (0.01)	0.02 (0.02)	0.39 (0.17)	0	0	0	0.42 (0.22)
Burkholderiales; Comamonadaceae; *Curvibacter*	0.07 (0.07)	0	0	0	0	0	0
Burkholderiales; Comamonadaceae; *Methylibium*	0.04 (0.03)	0.04 (0.02)	0	0	0	0	0
Burkholderiales; Comamonadaceae; *Ramlibacter*	0.07 (0.07)	0.03 (0.02)	0	0	0.63 (0.33)	0	0
Burkholderiales; Comamonadaceae; *Rubrivivax*	0.42 (0.21)	0.33 (0.11)	0	0	0	0	0
Burkholderiales; Comamonadaceae; *Variovorax*	0.08 (0.08)	0.06 (0.03)	0	3.81 (1.82)	0	0	0
Burkholderiales; Oxalobacteraceae; *Cupriavidus*	0.12 (0.08)	0.04 (0.04)	0	0	0	0	0
Burkholderiales; Oxalobacteraceae; *Ralstonia*	0.03 (0.02)	0.04 (0.03)	0	0	0	0	0
Rhodocyclales; Rhodocyclaceae; *Azoarcus*	0.08 (0.08)	0.02 (0.02)	0	0	0	0.40 (0.20)	0
**Deltaproteobacteria**							
Bdellovibrionales; Bdellovibrionaceae; *Bdellovibrio*	0	0.02 (0.02)	0	0	0	0	0
Myxococcales; Nannocystaceae; *Plesiocystis*	0	0.15 (0.10)	0	0	0	0	0
**Gammaproteobacteria**							
Legionellales; Coxiellaceae; *Aquicella*	0.19 (0.08)	0.16 (0.08)	0	0	0	0	0
Legionellales; Legionellaceae; *Legionella*	0.03 (0.03)	0.08 (0.07)	0	0	0	0	0
Oceanospirillales; Halomonadaceae; *Halomonas*	0.39 (0.09)	0.41 (0.15)	0.68 (0.36)	0	0.63 (0.43)	1.12 (0.70)	0
Pseudomonadales; Moraxellaceae; *Acinetobacter*	0.50 (0.37)	0.04 (0.04)	9.05 (4.79)	0.48 (0.48)	7.50 (3.11)	4.45 (3.70)	0
Pseudomonadales; Pseudomonadaceae; *Pseudomonas*	0.49 (0.03)	0.36 (0.18)	15.25 (6.37)	42.83 (9.16)	38.10 (9.61)	26.00 (4.82)	30.19 (3.26)
Xanthomonadales; Sinobacteraceae; *Alkanibacter*	0.22 (0.11)	0.12 (0.07)	0	0	0	0	0
Xanthomonadales; Sinobacteraceae; *Steroidobacter*	0.14 (0.06)	0.13 (0.06)	0	0	0	0	0
Xanthomonadales; Xanthomonadaceae; *Dokdonella*	0.03 (0.02)	0.02 (0.02)	0	0	0	0	0
Xanthomonadales; Xanthomonadaceae; *Lysobacter*	0.26 (0.09)	0.16 (0.07)	0	0	0	0	0
Xanthomonadales; Xanthomonadaceae; *Rhodanobacter*	0.73 (0.49)	0.21 (0.11)	0	0	0	0	0
Xanthomonadales; Xanthomonadaceae; *Stenotrophomonas*	0	0.02 (0.02)	0	1.38 (0.83)	1.48 (1.48)	0	0.80 (0.49)
Xanthomonadales; Xanthomonadaceae; *Thermomonas*	0.15 (0.06)	0.38 (0.22)	0	0	0	0	0
Xanthomonadales; Xanthomonadaceae; *Xanthomonas*	0	0	0.61 (0.40)	0	1.25 (0.75)	0	0
**Verrucomicrobia**							
Opitutae; Opitutales; Opitutaceae; *Opitutus*	0.03 (0.02)	0.12 (0.06)	0	0	0	0	0
Verrucomicrobiae; Verrucomicrobiales; Verrucomicrobiaceae; *Luteolibacter*	0.05 (0.05)	0.03 (0.02)	0	0	0	0	0
**Other phyla**							
Armatimonadetes; [Fimbriimonadia]; [Fimbriimonadales]; [Fimbriimonadaceae]; *Fimbriimonas*	0.85 (0.22)	0.75 (0.35)	0	0	0	2.40 (1.40)	0
Gemmatimonadetes; Gemmatimonadetes; Ellin5290	0.03 (0.02)	0.07 (0.07)	0	0	0	0	0
Gemmatimonadetes; Gemmatimonadetes; N1423WL	0.10 (0.07)	0.13 (0.07)	0	0	0	0	0
Nitrospirae; Nitrospira; Nitrospirales; 0319-6A21	0.11 (0.08)	0.05 (0.05)	0	0	0	0	0
Nitrospirae; Nitrospira; Nitrospirales; Nitrospiraceae; *Nitrospira*	1.21 (0.49)	0.94 (0.27)	0	0	0	0	0
Mollicutes; Anaeroplasmatales; Anaeroplasmataceae; *Asteroleplasma*	0	0	4.13 (3.15)	0	0	0	0

[a] Numbers in parenthesis are the standard errors ($n = 5$)
